# Motivations for a Career in Dentistry among Dental Students and Dental Interns in Kenya

**DOI:** 10.1155/2020/1017979

**Published:** 2020-07-29

**Authors:** Ochiba M. Lukandu, Lilian C. Koskei, Elizabeth O. Dimba

**Affiliations:** ^1^Department of Maxillofacial Surgery, Oral Medicine, Pathology and Radiology, School of Dentistry, Moi University, Eldoret, Kenya; ^2^Dental Department, Kericho County Referral Hospital, Kericho, Kenya; ^3^Department of Maxillofacial Surgery, Oral Medicine, Oral Pathology and Radiology, School of Dental Sciences, University of Nairobi, Nairobi, Kenya

## Abstract

A number of factors have been cited as determinants for choosing a career in dentistry around the globe. The purpose of this study was to determine motivations for a career in dentistry among dental students and dental interns in Kenya. This was a cross-sectional study where 293 individuals participated by filling and returning self-administered questionnaires. The mean age of all respondents was 22.3 years. Overall, 59.5% of the respondents had selected dentistry as their preferred career at the end of high school. Majority (76.1%) of the respondents agreed that personal interest in dentistry was an important motivating factor for them. This was followed closely by a desire to help or serve people (74%), a desire for a flexible work schedule (63%), and an aspiration to be self-employed (61.8%). There was no difference between males and females regarding these as motivating factors. On the other hand, among factors that the respondents felt had the lowest influence on their choice of dentistry was parental influence, where only 22% of the respondents indicated that this was a motivating factor for them. Other potential motivating factors such as influence by friends and siblings (30.3%) as well as career talk and guidance (41.3%) were also ranked low. In general, the respondents indicated that they were motivated much more by personal and humanitarian factors, when compared to financial and societal factors.

## 1. Introduction

There are many professions and career paths available in modern times, and it has become a challenge for an individual to make a choice regarding which career to pursue in life. A decision regarding which career to pursue in life has a huge impact on an individuals' future life. In Kenya, the first opportunity for a choice regarding one's future career comes just before and immediately after sitting examinations at the end of high school. The service is provided by the Ministry of Education through a central body that coordinates the placement of high school graduates into career programmes at various universities. The placement is based upon overall performance in examinations and also on individual career choices. Similar to many Asian [1] and other African [2] countries, a predetermined grade is used to enroll successful candidates into various degree programs. Medicine and dentistry are among programs that require very high grades for enrollment. More opportunities for choice or even change of choice of future career arise in the transition through college life and even during employment or episodes of unemployment. At every stage, a number of factors are thought to motivate or influence individuals to makes certain career choices.

Many factors have been cited as determinants for choosing a career within the medical field and dentistry in particular [3–9]. Dentistry is a noble profession providing essential health care to people and a great opportunity to meet new people on a regular basis. While career in dentistry may sound appealing to many people, it is important that those joining the profession have adequate information and are genuinely passionate about provision of oral health services [10]. Studies from several European countries have shown that most students who chose a career in dentistry were self-motivated [4]. Among self-motivating factors included desire and ability to help people, better opportunities for self-employment, and prestige. In India, even though economic and professional considerations were key factors influencing the choice of dentistry as a profession, many students cited influence from parents as a key motivation for their choice [5]. In the United Arab Emirates, aspirations for a reliable income in dentistry emerged as a key motivation among dental students [11]. This was particularly among male students, suggesting that motivating factors may vary with gender and culture.

There is limited information on determinants of career choices by students in developing nations, with only a few studies investigating career motivations and perceptions. Dentistry is a relatively young profession in Kenya, with only two out of over 65 universities providing undergraduate training in this field. In 2018, for a population of 48 million, the country had 1302 registered dentists with only 700 in active practice [12]. Dental training in Kenya takes five years at the dental school and an additional one-year internship training within selected hospitals. About 85% of dental students are publicly funded whereas the rest are privately funded. Privately funded students pay approximately 5000 USD per year. Both groups learn within the same dental schools under similar conditions. The country has postgraduate training opportunities in the fields of oral and maxillofacial surgery, pediatric dentistry, periodontology, and prosthodontics. In 2018, the country had only 147 dentists with specialized training, mostly in the fields of oral and maxillofacial surgery, pediatric dentistry, and restorative dentistry [12].

As the dental profession grows in Kenya, and more dental schools get started, career experts and dental educators will begin to take a keen interest in students' motivations for choosing a career in dentistry. The purpose of this study was to determine factors that motivate students to choose dentistry as a profession in Kenya. It was part of a larger study that also explored students' perceptions regarding career choice and dental training in Kenya as well as their long-term career expectation using both quantitative and qualitative methods.

## 2. Materials and Methods

The study was conducted among all undergraduate dental students and all newly graduated dentists (dentists on internship training) in Kenya. The study sites were the only two dental schools in the country, Moi University School of Dentistry (MU) and University of Nairobi School of Dental Sciences (UoN), as well as the only five dental internship training centers across the country. Ethical approval was granted by the Institutional Research Ethics Committee based in Eldoret, Kenya. Permission to conduct the study was also granted by the two dental training institutions. At the time of this study, there were 305 undergraduate dental students and 29 newly graduated dentists on internship training in the country, constituting a study population of 334. All these were eligible to participate and there were no exclusion criteria since this was a census study. Information about this study was sent out to the entire study population through their school and hospital administrations as well as class and group representatives.

A self-administered questionnaire was used to collect data. No identifying information was included on the questionnaire. The questionnaire was structured with both open ended and closed questions drawn from similar studies in other parts of the world. The questions were selected and designed to bring out potential extrinsic and intrinsic factors affecting choice of a career in dentistry. The questionnaire was divided into sections. The first section collected demographic information about the participants. The second section used a five-point Likert-type scale where the students were asked to indicate their level of agreement with statements outlining various factors that could have influenced their choice of dentistry as a career. There were fourteen factors selected to closely match factors investigated in similar studies across the globe. The purpose of the study was clearly explained to the participants and all were requested to sign consent forms prior to participation in the study. It took approximately 15 minutes to complete the questionnaire.

### 2.1. Statistical Analysis

All questionnaires were verified for completeness and data manually entered into a data analysis software (Statistical Package of Social Sciences version 22, IBM-SPSS, IL, USA). Descriptive statistics were used to determine percentages of responses regarding motivations and perceptions for career choice. Independent samples *t*-test was used to compare means of the ages of students from the two institutions. Analysis of categorical data was conducted using cross-tabulations with chi-square tests. A *p* value of less than 0.05 was considered significant.

## 3. Results

### 3.1. Demographics Information

Out of a total of 334 potential participants, 293 were available and took part in the study by filling and returning questionnaires, giving a total response rate of 87.7%. The response rate was 88.5% among respondents from University of Nairobi and 85.3% among respondents from Moi University. The lowest response rate was among fifth-year students at University of Nairobi (46.7%) and first-year students from Moi University (56.3%), both of whom were in preparation of examinations at the time of the study. The highest response rates (over 95%) were among third- and fourth-year respondents in both universities. There were 165 females accounting for 56.3% of the participants. Majority of the respondents (193) (65.9%) were from the University of Nairobi (Table 1). Mean age of all respondents was 22.3 years. The age of the respondents ranged from 18 to 31 with a mean of 21.8 among respondents from University of Nairobi and 18 to 33 with a mean of 23.3 among respondents from Moi University. There were a higher proportion of participants aged above 26 years among respondents from Moi University (18.7%) compared to only 3% among respondents from University of Nairobi. Upon further analysis, there was a significant difference in the mean ages between students at Moi University and those at University of Nairobi (*t*_154.93_ = 4.438, *p* < 0.001). The average age of Moi University students was 1.44 years older than the average age of students from University of Nairobi, with 95% CI [0.800, 2.08]. Regarding parents' occupation, majority (42.8%) of the respondents indicated that their parents worked in the financial sector (business, banking, and commerce), 18.7% indicated that their parents worked in education sector (mainly as teachers, lecturers, and education administrators) and 13.4% of the respondents indicated their parents were farmers (Table 1). Only 7% of the respondents indicated that their parents worked in the health sector (mainly as doctors and nurses).

### 3.2. Choice of Dentistry as a Profession

**Figure 1:**
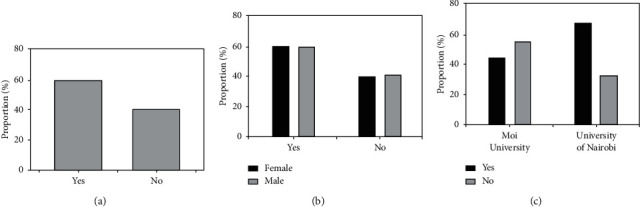
Preference of dentistry as a profession at the end of high school.

The respondents were asked to indicate whether they had selected dentistry as their preferred career at the end of high school education. In cases where dentistry was not their first choice, they were asked to indicate how they ranked it among other possible choices, and what career was their preferred choice. Overall, 59.5% (*n* = 172) of the respondents had selected dentistry as their preferred career at the end of high school (Figure 1(a)). There was no major difference between males (59%) and females (60%) in preference for dentistry (Figure 1(b)). However, the majority of students at the University of Nairobi (67.7%) had selected dentistry as their preferred choice compared to 45.5% of students at Moi University (Figure 1(c)). This difference was found to be significant upon further analysis using Chi square test, *X*^2^ (2, *N* = 288) = 15.64, *p* < 0.001.

**Figure 2:**
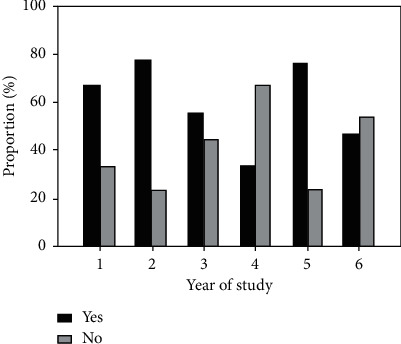
Variation in preference of dentistry as a career at the end of high school with level of study.

There was an obvious cyclic variation regarding those who had selected dentistry as their preferred choice with level of study ranging from a majority of 76.9% among second-year students to as low as 33.3% among fifth-year students in both universities (Figure 2). Regarding the time when the decision was made, 35.7% of the respondents who selected dentistry as their preferred choice indicated that they had made up their mind more than a year before the actual time of making the choice. This was twice as high when compared to the proportion (17.8%) among those who did not have dentistry as their preferred choice. Majority of respondents who did not have dentistry as their preferred choice indicated that they had it as their second choice (76.6%) while a small proportion (14%) did not have it as a choice at all. Respondents who did not have dentistry as their preferred choice had selected medicine (55.8%), engineering (25.0%), pharmacy (4.8%), and other professions (14.85) as their preferred choices. In hindsight, majority of the respondents agreed that they had made the right choice (80%, *n* = 225), compared to only 6% (*n* = 17) who were not content with the choice they had made.

### 3.3. Factors Influencing Choice of Dentistry

The respondents were asked to indicate by ranking their level of agreement with a statement that certain factors did influence their choice of a career in dentistry. Of the 14 potential factors studied, majority (76.1%) of the respondents agreed that personal interest in dentistry was an important motivating factor for them. This was followed closely by a personal desire to help or serve people (74%), desire to have a flexible work schedule (63%), and desire to be self-employed (61.8%) (Table 2). There was no difference between males and females regarding their choice of these as motivating factors. On the other hand, among factors that the respondents felt had the lowest influence on their choice of dentistry as a career was influence from their parents, where only 22% of the respondents agreed that this was a motivating factor for them. A slightly higher proportion of males (28.1%) than females (17.7%) indicated that persuasion by parents was a motivating factor. Only 11.9% of the respondents agreed that missing their preferred career choice was a factor in joining dental school. Other potential motivating factors such influence by friends and siblings (30.3%) as well as career talk and guidance (41.3%) were also ranked low.

**Figure 3:**
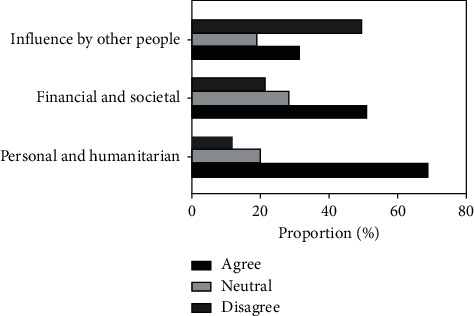
Motivations for choosing dentistry as a career.

To allow for further analysis, potential motivating factors were grouped broadly into three as follows: (1) influence by other people, (2) personal and humanitarian factors, and (3) financial and societal factors. In general, the respondents indicated that they were motivated much more by personal and humanitarian factors when compared to the other two groups of factors (Figure 3). The average agreement score for personal and humanitarian factors was 68.7%, whereas the average agreement score for financial and societal factors was 50.7%. The least ranked group of potential motivating factor was influence by other people which included influence by parents, siblings, career guides, and dentists, with an average agreement score of 31.4%.

## 4. Discussion

To the best of our knowledge, this is the first study in the Eastern Africa region to investigate motivations for the choice of dentistry as a career. The study was a census study where all available and willing members of the study population in Kenya took part. A response rate of over 87% was comparable to many similar studies across the globe [1, 5, 13]. The demographic findings in this study were also comparable to findings in studies conducted in many other countries [4, 14–16] including a slightly higher number of female students than male students and an average age of about 22 years. Students from Moi University were, on average, older than those from University of Nairobi by about one and a half years. This could be attributed to a higher proportion of students at Moi University who decided to join dentistry having already trained in other professions.

In a number of Asian countries including Japan [17] and Thailand [18], the occupation of family members appears to have an influence on career choices. In this study, only a small number (less than 10%) of the respondents had parents working within the health profession. Pursuing dentistry as part of family tradition in the field of health was therefore not a key factor among the respondents in this study. Two-thirds of the respondents had selected dentistry as their preferred career at the end of high school, with no gender difference in this respect. This is in contrast to findings in studies conducted in Nigeria (32%) [2] and India (38%) [17] where only about one-third of the students were found to have selected dentistry as their first career choice. The difference could be due to variations in the level of competitiveness for a career in dentistry as well as possible variations in the entry processes including entry examinations. [17].

A higher proportion of students at the University of Nairobi had selected dentistry as their preferred choice when compared to the proportion among students at Moi University. Possible reasons for this include the fact that the dental school at University of Nairobi is located in the capital city of the country and is a much larger, much older and well-known dental school having been established about 50 years ago whereas the one at Moi University is only about 10 years old. Students wishing to join dentistry would therefore prefer the former school.

The observed cyclic variation in proportion of those who had selected dentistry as their preferred career choice with level of study could partly be attributed to variation in student performance in university entry examinations from year to year. Given that more than one-third of those who had selected dentistry as their preferred career choice made up their mind more than one year before the actual selection, it is unlikely that factors such as career guidance and variations in national discourse on health did influence the cyclic variation. This issue should however be investigated in further studies.

A number of studies have shown that dentistry is often not the preferred career for most students undertaking dental training and that medicine is usually their preference [5, 10, 19]. These studies suggest that most students only end up in dental training because they fail to attain grades to allow them to join the more competitive medical programme. This is not supported by findings in this study since majority of respondents had dentistry as their preferred choice. However, it is worth noting that about one half of those who did not have dentistry as their preferred choice indicated that they had selected medicine as their preferred choice.

Findings in this study strongly point to personal interest in dentistry and personal desire to help or serve people as the most important motivating factors for the choice of a career in dentistry among dental students in Kenya. The desire to serve and help people and communities was found to be a key motivating factor among dental students in many developed countries including Sweden [20], Japan [21], UK [10], and even USA [22]. Self-motivation was also found to be a key motivation for a career in dentistry in Germany and Finland [13]. The finding that students in a developing country like Kenya considered factors such as personal interest and desire to help people more important than financial and economic factors was a notable variation from global trends.

Personal interest has also been ranked highly as a motivating factor in developing countries such as Iran [23], whereas prestige and helping others were found to be key motivating factors in Jordan [24] and Nigeria [2]. In Brazil [25], personal interest was considered a key motivating factor for many dental students, but helping others was not, even though it was shown to be of increasing importance over the years. In many developing countries, financial and economic factors tend to be ranked highly as motivations for a career in dentistry. The desire to secure a good job was found to be the most important motivating factor among dental students in South Africa [26]. In Nigeria [2], key motivation for choosing dentistry as a career was linked to a need to achieve personal goals such as job opportunities abroad, financial independence, and prestige. In China [21], dental students reported that their choice for a career in dentistry was mainly for financial reasons, and for prestige.

This study did not find any differences between males and females regarding their choice of motivating factors for a career in dentistry. Other motivating factors that were considered important were desire to have a flexible work schedule, and aspirations for self-employment. On the other hand, factors that had the lowest influence on choice of dentistry as a career were influence from parents and influence by friends and siblings as well as career talk and guidance. Influence by parents has been found to be an important factor why dental students in many Asian countries pursue a career in dentistry. In Japan, there are strong family connections within the dental fraternity, with a high number of dental students having parents who work as dentists [27]. In India, students were found to be highly influenced by their families in making important decisions, including career choice [5]. A possible explanation was suggested to be that, at that age, most of the students lived with their families. In this study, the respondents indicated that they were motivated much more by personal and humanitarian factors when compared to financial and societal factors. The least ranked group of potential motivating factors was influence by other people which included influence by parents.

## 5. Conclusion

In this study, it was found that dental students in Kenya were motivated much more by personal and humanitarian factors when compared to financial and societal factors. Influence by other people including influence by parents was ranked low as a motivating factor for a career in dentistry.

## Figures and Tables

**Table 1:** Demographic characteristics of the respondents.

Variable	Moi university	University of Nairobi	Total
Age in years	(*n* = 96)	(*n* = 193)	(*n* = 289)
Below 21	28	102	130
Between 22 and 25	50	85	135
Above 26	18	6	24

Gender	(*n* = 99)	(*n* = 193)	(*n* = 292)
Female	52	112	164
Male	47	81	128

Year of study	(*n* = 99)	(*n* = 193)	(*n* = 292)
Year 1	9	43	52
Year 2	13	53	66
Year 3	29	38	67
Year 4	21	32	53
Year 5	15	14	29
Internship	12	13	25

Parents' occupation	(*n* = 77)	(*n* = 166)	(*n* = 243)
Health	2	15	17
Education	24	22	46
Financial	20	84	104
Farmer	16	16	32
Others	15	29	44

**Table 2:** Motivational factors.

Motivation factor		Response in percentage		
Grouped factor	Individual factors	Agree	Neutral	Disagree
Personal/humanitarian	Personal interest	76.1	17.7	6.2
	Desire to serve/help people	74	18.5	7.6
	Flexible work pattern	63	16.8	20.2
	Desire for self-employment	61.8	25.8	12.4

Financial/societal	Desire for financial security	57.4	25.6	17.1
	Pride in title “doctor”	54	24.6	21.5
	Prestige/social status as dentist	40.7	34.6	24.7

Influence by others	Career talk/information	41.3	20.5	38.2
	Prior experience of treatment	37	15.8	47.3
	Prior exposure to dentistry	31.7	26.2	42.1
	Siblings'/friends' persuasion	30.3	17.1	52.6
	Family doctor	26	16.4	57.5
	Parents' persuasion	22	19.6	58.5

## Data Availability

The data used to support the findings of this study are included within the article. Raw data in an SPSS file are available upon request from the authors.

## Supplementary Materials

Supplementary Materials A copy of the questionnaire used for data collection in this study has been provided.
