# Supplementary material for: Motivations for a Career in Dentistry among Dental Students and Dental Interns in Kenya
Source: Int J Dent. 2020 Jul 29;2020:1017979. doi: 10.1155/2020/1017979 (PMC7411455; doi:10.1155/2020/1017979)
Supplement: Supplementary Materials — A copy of the questionnaire used for data collection in this study has been provided. [file 1017979.f1.pdf]

Questionnaire

MOTIVATION AND PERCEPTIONS ON CAREER CHOICE AND TRAINING AMONG UNDERGRADUATE DENTAL STUDENTS AND DENTAL INTERNS IN KENYA

SECTION A

Serial number…………

1. Demographic information
   a. Age …………………………………………….
   b. Gender ……………………………………………..
   c. Year of study ……………………………….………….….
   d. Institution of training ……………………………….……………
   e. Parents occupation …………………………………………….
2. Questions regarding motivation for choice of dentistry as a career

I chose dentistry because I was personally interested in the profession	◯ Strongly agree ◯ Agree ◯ Neutral ◯ Disagree ◯ Strongly disagree
The pride of calling myself/ earning title Doctor influenced my choice	◯ Strongly agree ◯ Agree ◯ Neutral ◯ Disagree ◯ Strongly disagree
Failure to be admitted in other programs led me to choose dentistry	◯ Strongly agree ◯ Agree ◯ Neutral ◯ Disagree ◯ Strongly disagree
Parents persuasion or compulsion played a major role in my choice	◯ Strongly agree ◯ Agree ◯ Neutral ◯ Disagree ◯ Strongly disagree
Siblings and other family members (other than parents) played a major role in my decision to join dentistry	◯ Strongly agree ◯ Agree ◯ Neutral ◯ Disagree ◯ Strongly disagree
Career talk/information was given to me prior to making my choice	◯ Strongly agree ◯ Agree ◯ Neutral ◯ Disagree ◯ Strongly disagree
Family dentist /doctor influenced me to choose dentistry	◯ Strongly agree ◯ Agree ◯ Neutral ◯ Disagree ◯ Strongly disagree
Desire for financial security influenced me to choose dentistry	◯ Strongly agree ◯ Agree ◯ Neutral ◯ Disagree ◯ Strongly disagree
Desire for flexible work pattern /less on call work /night duties led me to choose dentistry	◯ Strongly agree ◯ Agree ◯ Neutral ◯ Disagree ◯ Strongly disagree
Desire for self-employment after graduation led me to choose dentistry	◯ Strongly agree ◯ Agree ◯ Neutral ◯ Disagree ◯ Strongly disagree

Desire to help/serve people in my community influenced me to choose dentistry	Strongly agree — Agree — Neutral — Disagree — Strongly disagree
Prestige and social status of dentists made me chose dentistry influenced me to choose dentistry	Strongly agree — Agree — Neutral — Disagree — Strongly disagree
Prior exposure to dental/medical course influenced me to choose dentistry	Strongly agree — Agree — Neutral — Disagree — Strongly disagree
Prior experience of dental treatment influenced me to choose dentistry	Strongly agree — Agree — Neutral — Disagree — Strongly disagree

Duration of time choice was made/determined up to time of final choice

1. At time of making actual choice
2. Less than a month
3. Less than six months
4. Less than a year
5. Over a year

b. Dentistry was my first choice when making career choice (Y or N)…………………

c. If dentistry was not your first choice, what choice was it (2nd, 3rd, Not a choice at all) ……………………………………………………………………………………..…

d. If dentistry was not your first choice, which profession was?.....................................

Looking back from now, dentistry was the best choice to have made	Strongly agree — Agree — Neutral — Disagree — Strongly disagree

e. If not, what profession would you prefer now?..
